# Correction to: Patients in intensive care unit for COVID-19 pneumonia: the lung ultrasound patterns at admission and discharge. An observational pilot study

**DOI:** 10.1186/s13089-021-00219-5

**Published:** 2021-03-09

**Authors:** B. Paolo Persona, Ilaria Valeri, Francesco Zarantonello, Edoardo Forin, Nicolò Sella, Giulio Andreatta, Christelle Correale, Eugenio Serra, Annalisa Boscolo, Giovanni Volpicelli, Paolo Navalesi

**Affiliations:** 1 Institute of Anesthesia and Critical Care, Padua University Hospital, Via V. Gallucci, 13, 35121 Padova, Italy; 2 Anesthesia and Critical Care, Department of Medicine‑DIMED, University of Padua, Padua, Italy; 3 Department of Emergency Medicine, San Luigi Gonzaga University Hospital, Torino, Italy

## Correction to: Ultrasound J (2021) 13:10 https://doi.org/10.1186/s13089-021-00213-x

Following publication of the original article [1], we were notified that the first and last author names have been swapped.

The original article has been corrected.
